# Truncations in the amino terminus reveal a region key to supporting amphetamine-induced efflux by the human serotonin transporter

**DOI:** 10.1186/1471-2210-11-S2-A20

**Published:** 2011-09-05

**Authors:** Sonja Sucic, Carina Kern, Dragana Vidovic, Subhodeep Sarker, Oliver Kudlacek, Harald H Sitte, Michael Freissmuth

**Affiliations:** 1 Institute of Pharmacology, Center of Physiology and Pharmacology, Medical University of Vienna, 1090 Vienna, Austria

## Background

The serotonin transporter (SERT) terminates neurotransmission via reuptake of serotonin from the synaptic cleft. Upon stimulation with amphetamines, SERT switches into an outward transport mode to rapidly release serotonin. We have previously shown that truncation of the first 64 residues of SERT amino terminus leads to loss of amphetamine-induced efflux [1]. This was comparable to the effects of a single point mutation of a juxtamembrane threonine residue at position 81 [1].

## Methods

Truncation mutants of SERT amino terminus were generated by removing 22 (Δ22-SERT), 32 (Δ32-SERT) or 42 (Δ42-SERT) amino terminal residues. In addition, alanine scanning mutagenesis was performed along a segment of amino acid residues 32–42. All mutants were pharmacologically characterised in uptake, binding and efflux studies.

## Results

Cellular localisation of the mutants examined by confocal microscopy, revealed no differences compared to the wild-type SERT. Functional analysis showed only modest changes in their substrate uptake properties (no significant changes in the K_m_ values and a moderate decrease in the V_max_ value of Δ42-SERT). Similarly, there were no marked alterations in the K_D_ and B_max_ values of imipramine or in the K_i_ values of *p*-chloroamphetamine and ibogaine, determined in radiolabelled imipramine binding assays. However, while amphetamine-induced efflux was unimpaired for Δ22-SERT and to a slight extent decreased for Δ32-SERT, it was completely abolished for Δ42-SERT.

## Conclusions

Our results shed new light on the functional role of the amino terminus and point to the segment encompassing residues 32–42 as a region of key importance to supporting amphetamine-induced efflux by SERT.
